# Live Probiotic *Lactobacillus johnsonii* BS15 Promotes Growth Performance and Lowers Fat Deposition by Improving Lipid Metabolism, Intestinal Development, and Gut Microflora in Broilers

**DOI:** 10.3389/fmicb.2017.01073

**Published:** 2017-06-12

**Authors:** Hesong Wang, Xueqin Ni, Xiaodan Qing, Dong Zeng, Min Luo, Lei Liu, Guangyao Li, Kangcheng Pan, Bo Jing

**Affiliations:** ^1^Animal Microecology Institute, College of Veterinary, Sichuan Agricultural UniversityChengdu, China; ^2^Ya’an Agricultural Science and Technology Development Co., Ltd.Ya’an, China

**Keywords:** *Lactobacillus*, lipid metabolism, gut microbiota, probiotic, broiler

## Abstract

Numerous studies have focused on the beneficial effects of probiotics in animals. Even so, additional information should be obtained about the mechanisms by which a useful probiotic strain successfully exerts such beneficial effects. In this study, we evaluated the effect of the dietary supplementation of both live and disrupted *Lactobacillus johnsonii* (LJ) strain BS15 in broilers at different ages. Specifically, growth performance, lipid metabolism, gut microbiota, intestinal development, and digestive ability of the broilers were assessed. A total of 180 1-day-old Cobb 500 chicks were randomly distributed into three groups. These chicks were fed diets supplemented with 1 × 10^6^ colony-forming units (cfu) LJ per gram of feed (LJ group); 1 × 10^6^ cfu disrupted LJ per gram of feed (D-LJ group); and de Man, Rogosa, and Sharpe liquid medium (control group), respectively, throughout a 42-day experimental period. The results demonstrated that LJ supplementation of feed had a positive effect on the average daily gain and starter feed conversion ratio. In addition, LJ supplementation of feed decreased serum triglyceride and low-density lipid cholesterol levels, as well as abdominal fat deposition. LJ also reduced the mRNA levels of lipoprotein lipase in adipose tissue and stearoyl-CoA desaturase-1 in the liver. LJ diminished the mRNA quantities of the sterol regulatory element binding protein-1c and fatty acid synthase, as well as increased the level of serum high-density lipid cholesterol. LJ increased the mRNA quantities of peroxisome proliferator-activated receptor α, acyl-CoA oxidase in the liver, and carnitine palmitoyltransferase-1. LJ also improved the intestinal development and digestive ability mainly by increasing the villus height/crypt depth ratio in the ileum. The probiotic increased the levels of epidermal growth factor and insulin-like growth factor-1, as well as the activities of trypsin and lipase in the jejunum and ileum. LJ exerted beneficial effects on the intestinal flora. Specifically, LJ markedly enhanced the population of Bacteroidetes and *Lactobacillus* spp. Moreover, the probiotic reduced the population of Enterobacteriaceae and the Firmicutes/Bacteroidetes ratio. Slight changes caused by disrupted LJ were detected. These findings indicated that live LJ supplementation may promote growth performance and lower fat deposition in broilers.

## Introduction

Given the ban on in-feed antibiotic growth promoter usage in Europe and the global trend to reduce the utilization of antibiotics as growth promoters in livestock diets, determining alternatives to the utilization of antibiotic growth promoters has attracted increasing attention (Van et al., 2009). Probiotics, live cultures of bacteria provided to benefit the host are one of the alternative treatment strategies that can be used (Upadhaya et al., 2016). To date, a number of probiotics have been used in the poultry industry. As extensively used probiotics in the poultry industry, *Lactobacillus* strains can promote growth performance (Nakphaichit et al., 2011; Shen et al., 2014), improve meat quality (Keokamnerd et al., 2007; Lv et al., 2012), enhance immune response (Huang et al., 2004; Stringfellow et al., 2011), and prevent some avian diseases (Pascual et al., 1999; Yamazaki et al., 2012; Youn et al., 2012). However, it has proved difficult to demonstrate the mechanism whereby probiotics affect the gastrointestinal tract because probiotic strains exert their beneficial effects via different mechanisms in which other microbiota may be involved as well (Rubio et al., 2014; Serban, 2014; Patten and Laws, 2015). One probiotic strain may exhibit properties and clinical effects relatively different from another, even when the strains belong to the same bacterial species (Vieira et al., 2013). In addition, previous studies demonstrated the difficulty of reliably extrapolating the *in vitro* influences of probiotics to the *in vivo* situation (Jacobsen et al., 1999). Although numerous studies have focused on the positive effects of probiotics in animals, the mechanisms by which probiotics can successfully exert beneficial effects remain unclear. Therefore, the practical value in gaining further *in vivo* information about a useful probiotic strain should not be ignored. For example, it is important to find out whether or not live microorganisms are needed to induce the benefits.

*Lactobacillus johnsonii* BS15 (CCTCC M2013663) was isolated from homemade yogurt collected from Hongyuan Prairie, Aba Autonomous Prefecture, China. This probiotic strain can attenuate inflammation and mitochondrial injury, improve gut environment, and prevent non-alcoholic fatty liver disease in obese mice (Xin et al., 2014). In a previous study in broilers, *L. johnsonii* BS15 (LJ) supplementation improved growth performance and significantly enhanced the meat’s nutritional value, including the fatty acid composition, especially the polyunsaturated fatty acid content (Liu et al., 2016). However, the information on how LJ exerts its beneficial effects in broilers is limited. Thus, the present study aimed to assess the effects of LJ on growth performance, lipid metabolism, gut microbiota, intestinal development, and digestive ability. This study also aimed to examine whether the effects of LJ are related to the living state of microorganisms. The latter goal was achieved by comparing the results from the addition of live and disrupted bacterial cells.

## Materials and Methods

### Animals, Treatment, and Sampling

A total of 180 1-day-old male chicks (Cobb 500) with similar body weight were purchased from Chia Tai broiler hatchery (Chengdu, China). Chicks were weighed and divided into three treatment groups. Each group consisted of six replicates with 10 birds per replicate at the Key Laboratory of Animal Disease and Human Health of Sichuan Province, Sichuan Agricultural University. Birds were fed *ad libitum* and given free access to water throughout the entire experiment. Artificial light was provided 24 h per day by fluorescent light. The room temperature was maintained at 33°C for the first 3 days. Afterward, the temperature was gradually reduced by 3°C weekly until 24°C. Then, the room temperature was maintained at 24°C until the end of the experiment. The starter and finisher diet formulas are shown in **Table 1**. All the diets were formulated to satisfy or exceed the National Research Council [NRC] (1994) requirements for broilers. The three bird groups were fed with diets in mash form as follows: control group (basal diet + de Man, Rogosa, and Sharpe [MRS] liquid medium), LJ group (basal diet + 1 × 10^6^ cloning-forming units [cfu] LJ/g as fed), and D-LJ group (basal diet + 1 × 10^6^ cfu disrupted LJ/g as fed [>95% LJ disrupted]). Chicks were weighed, and feed intake was recorded on the morning of days 21 and 42. The average daily gain (ADG), average daily feed intake (ADFI), and feed conversion ratio (FCR) were also calculated. All animal experiments were performed in accordance with the guidelines for the care and use of laboratory animals approved by the Institutional Animal Care and Use Committee of Sichuan Agricultural University (approval number: SYXKchuan2014-187).

**Table 1:** Composition of basal diets for broiler chickens.

Ingredient^1^	Starter diet (%) 1–21 days	Finisher diet (%) 22–42 days
Ground yellow corn	56.00	59.50
Soybean meal	37.00	32.90
Soybean oil	3.66	4.70
Ground limestone	0.57	0.50
Dicalcium phosphate	1.80	1.60
Salt	0.30	0.30
Choline chloride	0.10	0.10
DL-Met	0.24	0.12
Micronutrients^2^	0.33	0.33
**Calculated nutrients level**		
ME (MJ kg^-1^)	12.40	12.80
CP	21.20	19.70
Lys	1.19	1.08
Met	0.50	0.40
Met + Cys	0.86	0.74
Ca	0.85	0.77
Non-phytate P	0.44	0.40

*^1^The ingredient and nutrient composition are reported on an as-fed basis.*

*^2^Micronutrients were provided per kilogram of diet: vitamin A (all-*trans* retinol acetate), 12,500 IU; cholecalciferol, 2,500 IU; vitamin E (all-rac-a-tocopherol acetate), 18.75 IU; vitamin K (menadione Na bisulfate), 5.0 mg; thiamine (thiamine mononitrate), 2.5 mg; riboflavin, 7.5 mg; vitamin B6, 5.0 mg; vitamin B12, 0.0025 mg; pantothenate, 15 mg; niacin, 50 mg; folic acid, 1.25 mg; biotin, 0.12 mg; Cu (CuSO_*4*_⋅5H_*2*_O), 10 mg; Mn (MnSO_*4*_⋅H_*2*_O), 100 mg; Zn (ZnSO_*4*_⋅7H_*2*_O), 100 mg; Fe (FeSO_*4*_⋅7H_*2*_O), 100 mg; I (KI), 0.4 mg; and Se (Na_*2*_SeO_*3*_), 0.2 mg.*

On the mornings of days 21 and 42, six birds (one bird per cage) of each treatment were randomly selected. Blood samples from the wing vein were collected. Serum samples were obtained from these blood samples by incubation at 4°C for 30 min and subsequent centrifugation at 1,500 × *g* for 20 min. The same birds were sacrificed by exsanguination under anesthesia in accordance with the institutional animal care guidelines. The abdominal fat pad from the proventriculus (surrounding the gizzard and running down to the cloaca) was removed and weighed. The abdominal fat percentage was calculated (a proportion of total body weight). Subsequently, a small sample each from the adipose tissue, liver, and small intestine (including content and epithelial tissue of the duodenum, jejunum, and ileum) were removed and washed with ice-cold sterilized saline. These samples were then frozen in liquid nitrogen and stored at -80°C for subsequent analysis of enzyme activities and/or gene expression. Part of each small intestinal section (1 cm^2^) was fixed in 4% formalin solution for villus morphology measurement. Total liver and adipose tissue RNA were extracted using RNAiso Plus (TaKaRa, Dalian, China) in accordance with the manufacturer’s guidelines. The quality and quantity of isolated RNA were assessed from the ratio of the absorbance values at 260 and 280 nm and agarose gel electrophoresis. First-strand complementary DNA (cDNA) was synthesized from 1 μg of total RNA using a PrimeScript^TM^ RT reagent kit with gDNA Eraser (TaKaRa, Dalian, China). All the cDNA products were frozen at -20°C until further use. A sample of ileal content (500 mg each) was immediately removed and dipped in liquid nitrogen. Genomic DNA was isolated from the ileum samples (200 mg each) using a TIANamp stool DNA kit (Tiangen, Beijing, China) in accordance with the manufacturer’s instructions. DNA quality was analyzed by 2% (w/v) agarose gel electrophoresis. Finally, DNA was stored at -80°C until further analysis.

### Feed Preparation

**FIGURE 1:**
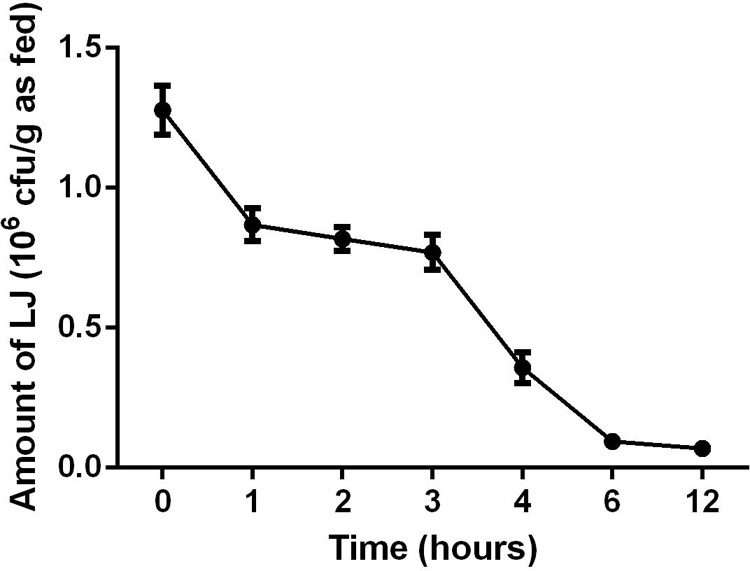
Changes in *Lactobacillus johnsonii* BS15 amount from 0 to 12 h. Data are presented as mean ± standard deviation (*n* = 5).

The viable counts of LJ cell preparations were evaluated by heterotrophic plate counts after maintaining cultures in MRS broth at 37°C for 36 h under an anaerobic environment. A portion of cells was disrupted (20–40 intensity; three times; 1 min per time; volume: 5 ml; cooling:ice-bath) using an ultrasonic processor-UH500A (Autoscience, Tianjin, China) prior to supplementation. Supplementation was performed before each feeding. The basic procedures were as follows: Approximately 10 mL of LJ solution/disrupted LJ solution/MRS liquid medium (diluted with the same amount of PBS) was thoroughly mixed with 1000 g of diet. Each feed amount was consumed by the chicks within 3 h and resupplied every 3 h. The number of viable bacteria in the diet was altered over time (**Figure 1**).

### Hormonal and Biochemical Serum Evaluations

Insulin (INS), growth hormone (GH), leptin (LEP), free triiodothyronine (FT3), and free thyroxine (FT4) levels in the serum were determined with commercial RIA kits in accordance with the manufacturer’s instructions. The kits were purchased from Beijing North Institute of Biological Technology (Beijing, China). Aspartate aminotransferase (AST) and alanine aminotransferase (ALT) were determined with the commercial kits (microplate method) obtained from Nanjing Jiancheng Bioengineering Institute (Nanjing, Jiangsu, China). The contents of triglycerides (TG), total cholesterol (TC), high-density lipid cholesterol (HDL-C), and low-density lipid cholesterol (LDL-C) in serum were determined using a GS200 Automatic Biochemical Analyzer (Shenzhen Genius Electronics Co., Ltd., Shenzhen, China) in accordance with the manufacturer’s instructions.

### Real-Time Quantitative PCR (qPCR) Analysis of Gene Expression

Using the prepared cDNA products of liver and adipose tissue, PCRs were performed using a CFX96 Real-Time PCR Detection System (Bio-Rad, Hercules, CA, United States) with a SYBR Premix Ex Taq^TM^ PCR kit (TaKaRa, Dalian, China). The thermocycle protocol lasted for 5 min at 95°C, followed by 40 cycles of 15 s denaturation at 95°C, and 30 s annealing/extension at optimum temperature (**Table 2**). A final melting curve analysis was used to monitor the purity of the PCR product. The primer sequences for the targeted genes are presented in **Table 2**. Standard curves were obtained from serial dilutions of samples. The _ΔΔ_*C*t method was used to estimate mRNA abundance. *C*t is calculated as (*C*t_target_ -*C*t_GAPDH_)_treatment_ - (*C*t_target_ -*C*t_GAPDH_)_control_, where GAPDH is glyceraldehyde-3-phosphate dehydrogenase. Relative gene expression levels were normalized to those of the eukaryotic housekeeping gene GAPDH. All samples (*n* = 6) in each group were analyzed in triplicate. The mean values of these measurements were used to calculate the mRNA expression levels of peroxisome proliferator-activated receptor γ (PPARγ), adipose TG lipase (ATGL), and lipoprotein lipase (LPL) in adipose tissue. Likewise, the measured values were also used to calculate the mRNA expression levels of the sterol regulatory element binding protein-1c (SREBP-1c), fatty acid synthase (FAS), stearoyl-CoA desaturase-1 (SCD1), acetyl-CoA carboxylase (ACC), PPARα, carnitine palmitoyltransferase-1 (CPT-1), and acyl-CoA oxidase (ACOX) in the liver.

**Table 2:** Gene-specific primers of lipid metabolism-related enzymes.

Gene name^1^	Primer sequence (5→3)	Tm (°C)/size (bp)	Accession
SREBP-1c	F:GAGGAAGGCCATCGAGTACA R:GGAAGACAAAGGCACAGAGG	60.3/220	AY029224
FAS	F:CTATCGACACAGCCTGCTCCT R:CAGAATGTTGACCCCTCCTACC	62.0/107	J03860
SCD1	F:ACCATACATTCCCCTACGACT R:TTTTCCGGGCCAAGATGACC	56.0/144	NM204890
ACC	F:AATGGCAGCTTTGGAGGTGT R:TCTGTTTGGGTGGGAGGTG	60.9/136	NM205505
PPARα	F:TGGACGAATGCCAAGGTC R:GATTTCCTGCAGTAAAGGGTG	60.3/813	AF163809
CPT-1	F:CAATGAGGTACTCCCTGAAA R:CATTATTGGTCCACGCCCTC	57.5/337	AY675193
ACOX	F:ATGTCACGTTCACCCCATCC R:AGGTAGGAGACCATGCCAGT	54.0/133	NM001006205
PPARγ	F:CCAGCGACATCGACCAGTT R:GGTGATTTGTCTGTCGTCTTTCC	57.5/145	AF163811
ATGL	F:TCCTTCACCTTCAGCGTCCA R:AGTGTTGTCCTCCATCTGGTC	54.0/113	EU852334
LPL	F:CAGTGCAACTTCAACCATACCA R:AACCAGCCAGTCCACAACAA	60.0/150	NM205282
GADPH	F:GGTGAAAGTCGGAGTCAACGG R:CGATGAAGGGATCATTGATGGC	58.4/108	NM204305

*^1^SREBP-1c, sterol regulatory element binding protein-1c; FAS, fatty acid synthase; SCD1, stearoyl-CoA desaturase-1; ACC, acetyl-CoA carboxylase; PPARα, peroxisome proliferator-activated receptor α; CPT-1, carnitine palmitoyltransferase-1; ACOX, acyl-CoA oxidase; PPARγ, peroxisome proliferator-activated receptor γ; ATGL, adipose triglyceride lipase; LPL, lipoprotein lipase; GADPH, glyceraldehyde-3-phosphate dehydrogenase.*

### Villus Morphology Measurement

After rinsing with water, the small intestinal samples in 4% formalin solution were dehydrated in a graded series of absolute ethanol (50, 70, 80, 90, and 100%), cleared with benzene twice, and saturated with and embedded in paraffin. Sections with 5 μm thickness were stained with hematoxylin and eosin and observed by light microscopy. The small intestinal villus height (Vh) and crypt depth (Cd) were determined using an image analysis program (Image-Pro Plus 6.1, Rockville, MD, United States), and the Vh/Cd ratio was calculated.

### Enzyme Activity Evaluation of the Small Intestine

The epithelial INS-like growth factor-1 (IGF-1) and epidermal growth factor (EGF) contents in the small intestine were quantified using enzyme-linked immunosorbent assay kits specific for chicken (RD Ltd., United States). The contents were determined by standard curve and expressed in nanograms per milliliter. Amylase, trypsin, and lipase activities of the intestinal contents were measured using the commercial kits of Nanjing Jiancheng Bioengineering Institute (Nanjing, Jiangsu, China).

### qPCR Quantification of Intestinal Microbial Content

A CFX Connect^TM^ Real-Time system (Bio-Rad, Hercules, CA, United States) and SYBR^®^ Premix Ex Taq^TM^ II (TaKaRa, Dalian, China) were used to perform qPCR. The population of total bacteria, Firmicutes, Bacteroidetes, *Lactobacillus* spp. and Enterobacteriaceae in the ileum was estimated. The primers for the qPCR of the microflora are listed in **Table 3**. The reaction mixture (25 μL) included sterile deionized water (9.5 μL), SYBR^®^ Premix Ex Taq^TM^ II (12.5 μL), forward and reverse primers (1 μL), and DNA template (1 μL). The PCR program was as follows: 95°C for 1 min, subsequent 40 cycles of 94°C for 15 s, and annealing at optimal temperatures for 30 s and 72°C for 30 s. Melting curves were generated to regulate the specificity of the PCR primers. The population of *Lactobacillus* spp., Bacteroidetes, Firmicutes, and Enterobacteriaceae was evaluated, as described by Xin et al. (2014).

**Table 3:** Primer information on the microflora for qPCR.

Target species	Primer sequence (5→3)	Tm (°C)/size (bp)
Total bacteria	F: CGGYCCAGACTCCTACGGG R: TTACCGCGGCTGCTGGCAC	60.0/130
Firmicutes	F: GGAGYATGTGGTTTAATTCGAAGCA R: AGCTGACGACAACCATGCAC	64.5/126
Bacteroidetes	F: GGARCATGTGGTTTAATTCGATGAT R: AGCTGACGACAACCATGCAG	60.0/126
*Lactobacillus* spp.	F: AGCAGTAGGGAATCTTCCA R: CACCGCTACACATGGAG	64.5/341
Enterobacteriaceae	F: CATTGACGTTACCCGCAGAAGAAGC R: CTCTACGAGACTCAAGCTTGC	62.5/195

### Statistical Analysis

All results were expressed as means ± standard deviation and analyzed by one-way ANOVA. Duncan’s multiple-range test was used for multiple comparison when a significant interaction was detected. All the statistical analyses were conducted using SigmaPlot for Social Sciences version 12. Differences at *P* < 0.05 were considered statistically significant. Data on growth performance were based on a cage basis (*n* = 6), whereas the other data were based on individual broilers (six replicates of one chick per cage).

## Results

### Growth Performance

The growth performance results are shown in **Table 4**. The LJ group showed a higher (*P* < 0.05) ADG and a slightly lower FCR (*P* = 0.099) in the starter phase (1–21 days) than those of the control and D-LJ groups. High ADFI and ADG (*P* < 0.05) were also observed in the LJ group in the finisher phase (22–42 days) and the entire period (1–42 days) of the experiment. However, the ADFI of the starter phase, the FCR of the finisher phase, and the overall FCR were not improved by LJ addition (*P* > 0.05). Similarly, no significant difference was observed between the growth performance of the control and D-LJ groups (*P* > 0.05).

**Table 4:** Effects of *L. johnsonii* BS15 on chicken growth performance^1^.

Parameter^2^	Control	LJ	D-LJ
**Days 1–21**
ADFI, g/day	52.26 ± 2.51	53.75 ± 2.05	52.79 ± 2.05
ADG, g/day	33.53 ± 1.37^b^	36.68 ± 1.49^a^	34.01 ± 1.50^b^
FCR	1.56 ± 0.07	1.47 ± 0.10	1.55 ± 0.06
**Days 22–42**			
ADFI, g/day	159.80 ± 4.51^b^	168.21 ± 3.17^a^	158.56 ± 4.84^b^
ADG, g/day	78.73 ± 2.04^b^	84.11 ± 3.21^a^	78.80 ± 1.24^b^
FCR	1.99 ± 0.04	2.00 ± 0.08	2.01 ± 0.05
**Overall (day 1 to 42)**			
ADFI, g/day	104.53 ± 2.00^b^	110.98 ± 2.08^a^	105.68 ± 3.20^b^
ADG, g/day	56.13 ± 0.97^b^	60.40 ± 2.09^a^	56.40 ± 1.00^b^
FCR	1.86 ± 0.03	1.84 ± 0.08	1.87 ± 0.04

*^1^Means in the same row with no superscript or with a common superscript letter do not differ significantly (*P* < 0.05). Data are presented as mean ± standard deviation (*n* = 6; a cage was regarded as one experimental unit). Control group = basal diet without supplementation; LJ group = basal diet with 1.0 × 10^6^ cfu LJ/g diet as fed; D-LJ group = basal diet with 1.0 × 10^6^ cfu disrupted LJ/g diet as fed.*

*^2^ADFI, average daily feed intake; ADG, average daily gain; FCR, feed conversion ratio (ADFI/ADG).*

### Hormonal and Biochemical Serum Evaluations

The results in **Table 5** show no difference in serum hormonal level among the three experimental groups at days 21 and 42. Results of biochemical parameters can also be found in **Table 6**. None of the parameters were influenced by LJ at day 21 (*P* > 0.05). Although the AST, ALT, and TC levels were not altered (*P* > 0.05) by LJ, higher levels of HDL-C (*P* < 0.05) and lower levels of TG and LDL-C (*P* < 0.05) were observed in the LJ group in comparison with the control and D-LJ groups. The D-LJ treatment exerted no influence on the hormonal and biochemical parameters because no differences were detected (*P* > 0.05) between the D-LJ and control groups.

**Table 5:** Effects of *L*. *johnsonii* BS15 on serum hormone levels^1^.

Parameter^2^	Control	LJ	D-LJ
**Day 21**			
INS, μIU/mL	3.29 ± 0.10	3.20 ± 0.23	3.09 ± 0.25
GH, ng/mL	1.08 ± 0.09	1.08 ± 0.09	1.05 ± 0.05
LEP, ng/mL	0.53 ± 0.03	0.55 ± 0.04	0.54 ± 0.02
FT3, ng/mL	5.52 ± 0.51	5.59 ± 0.36	5.66 ± 0.38
FT4, ng/mL	10.07 ± 1.41	10.21 ± 0.71	10.60 ± 0.99
**Day 42**			
INS, μIU/mL	3.13 ± 0.14	3.32 ± 0.23	3.29 ± 0.22
GH, ng/mL	1.11 ± 0.10	1.12 ± 0.10	1.10 ± 0.08
LEP, ng/mL	0.36 ± 0.03	0.34 ± 0.04	0.35 ± 0.04
FT3, ng/mL	3.63 ± 0.27	3.40 ± 0.20	3.55 ± 0.05
FT4, ng/mL	13.79 ± 1.13	13.55 ± 1.22	13.75 ± 0.48

*^1^Means in the same row with no superscript or with a common superscript letter do not differ significantly (*P* < 0.05). Data are presented as mean ± standard deviation (for six replicates of one chick per cage). Control group = basal diet without supplementation; LJ group = basal diet with 1.0 × 10^6^ cfu LJ/g diet as fed; D-LJ group = basal diet with 1.0 × 10^6^ cfu disrupted LJ/g diet as fed.*

*^2^INS, insulin; GH, growth hormone; LEP, leptin; FT3, free triiodothyronine; FT4, free thyroxine.*

**Table 6:** Effects of *L*. *johnsonii* BS15 on serum biochemical parameters^1^.

Parameter^2^	Control	LJ	D-LJ
**Day 21**			
AST, IU/L	137.62 ± 6.66	132.58 ± 10.21	138.40 ± 9.15
ALT, IU/L	11.33 ± 1.74	12.15 ± 1.24	12.09 ± 1.35
TG, mmol/L	0.29 ± 0.03	0.31 ± 0.04	0.30 ± 0.06
TC, mmol/L	2.65 ± 0.17	2.63 ± 0.12	2.60 ± 0.24
HDL-C, mmol/L	0.49 ± 0.09	0.46 ± 0.08	0.49 ± 0.06
LDL-C, mmol/L	2.34 ± 0.21	2.38 ± 0.16	2.36 ± 0.21
**Day 42**			
AST, IU/L	126.79 ± 11.36	133.23 ± 6.46	134.70 ± 6.33
ALT, IU/L	12.46 ± 1.42	12.85 ± 1.49	11.87 ± 0.95
TG, mmol/L	0.41 ± 0.05^a^	0.31 ± 0.03^b^	0.35 ± 0.04^a^
TC, mmol/L	2.55 ± 0.21	2.57 ± 0.28	2.63 ± 0.23
HDL-C, mmol/L	0.48 ± 0.06^b^	0.59 ± 0.04^a^	0.52 ± 0.09^b^
LDL-C, mmol/L	1.93 ± 0.08^a^	1.71 ± 0.07^b^	1.86 ± 0.13^a^

*^1^Means in the same row with no superscripts or with a common superscript letter do not differ significantly (*P* < 0.05). Data are presented as mean ± standard deviation (for six replicates of one chick per cage). Control group = basal diet without supplementation; LJ group = basal diet with 1.0 × 10^6^ cfu LJ/g diet as fed; and D-LJ group = basal diet with 1.0 × 10^6^ cfu disrupted LJ/g diet as fed.*

*^2^AST, aspartate aminotransferase; ALT, alanine aminotransferase; TG, triglyceride; TC, total cholesterol; HDL-C, high-density lipoprotein cholesterol; LDL-C, low-density lipoprotein cholesterol.*

### Fat Deposition and Gene Expression of Lipid Metabolism-Related Enzymes in Adipose Tissue

**FIGURE 2:**
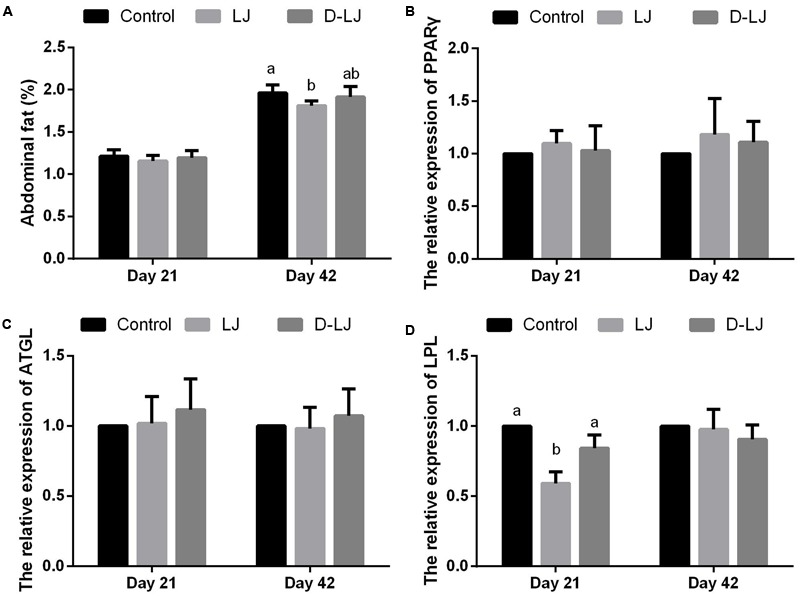
Fat deposits and relative mRNA expression levels of lipid metabolism-related enzymes in adipose tissue. Bars with different letters are significantly different on the basis of Duncan’s multiple range tests (*P* < 0.05). Data are presented as mean ± standard deviation (six replicates of one chick per cage). **(A)** Abdominal fat percentage; **(B–D)** relative expression of PPARγ, ATGL, and LPL, respectively. PPARγ, peroxisome proliferator-activated receptor γ; ATGL, adipose triglyceride lipase; LPL, lipoprotein lipase.

The abdominal fat percentage in the LJ group was evidently lower (*P* < 0.05) than that in the control group (**Figure 2A**). Such percentage was also lower than that in the D-LJ group, but the difference was not significant (*P* > 0.05). The gene expression results related to lipid metabolism, specifically, those of the enzymes in adipose tissue, are presented in **Figures 2B–D**. The PPARγ and ATGL showed no significant alterations (*P* > 0.05) at both days 21 and 42 (**Figures 2B,C**). The LPL (*P* > 0.05) also showed no evident difference among the three groups at day 42 (**Figure 2D**). Nevertheless, the gene expression of LPL in the LJ group at day 21 was significantly decreased in comparison to the control and D-LJ groups (**Figure 2D**).

### Gene Expression of Lipid Metabolism-Related Enzymes in the Liver

**FIGURE 3:**
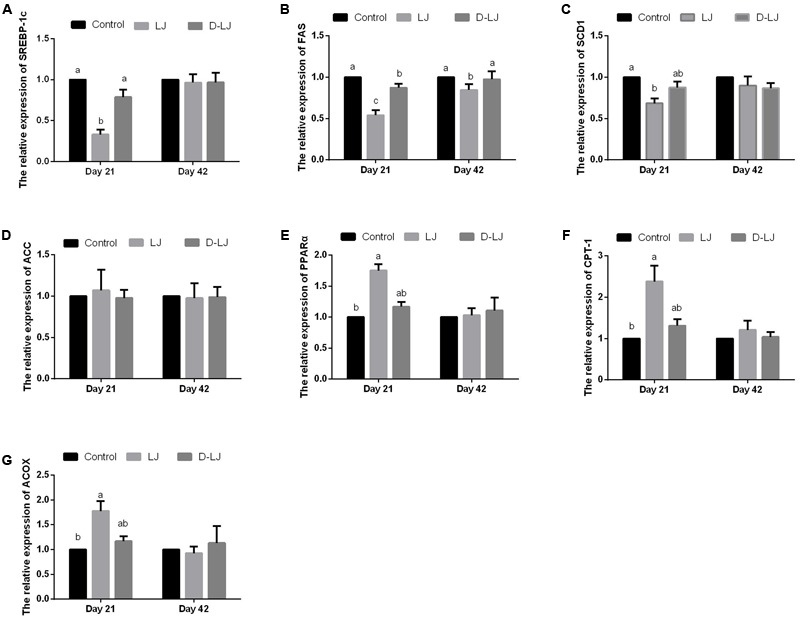
Relative mRNA expression levels of lipid metabolism-related enzymes in the liver. Bars with different letters are significantly different in accordance with Duncan’s multiple range tests (*P* < 0.05). Data are presented as mean ± standard deviation (six replicates of one chick per cage). **(A–G)** Relative expression of SREBP-1c, FAS, SCD1, ACC, PPARα, CPT-1, and ACOX, respectively. SREBP-1c, sterol regulatory element binding protein-1c; FAS, fatty acid synthase; SCD1, stearoyl-CoA desaturase-1; ACC, acetyl-CoA carboxylase; PPARα, peroxisome proliferator-activated receptor α; and CPT-1, carnitine palmitoyltransferase-1; ACOX, acyl-CoA oxidase.

Upregulated PPARγ, CPT-1, and ACOX gene expression levels and downregulated SREBP-1c, FAS, and SCD1 gene expression levels were observed at day 21 in the LJ group relative to those in the control (*P* < 0.05) and D-LJ groups (*P* < 0.05 for SREBP-1c and FAS) (**Figure 3**). Furthermore, the FAS gene expression in the D-LJ group was significantly lower than that in the control group. However, except for the FAS gene expression, which was significantly lower (*P* < 0.05) in the LJ group (**Figure 3B**), none of the gene expression levels of the lipid metabolism-related enzymes was changed (*P* > 0.05) with LJ addition at 42 days. D-LJ addition also exerted limited changes (*P* > 0.05), especially at day 21 (**Figure 3**).

### Morphometric Analysis of the Small Intestine

**FIGURE 4:**
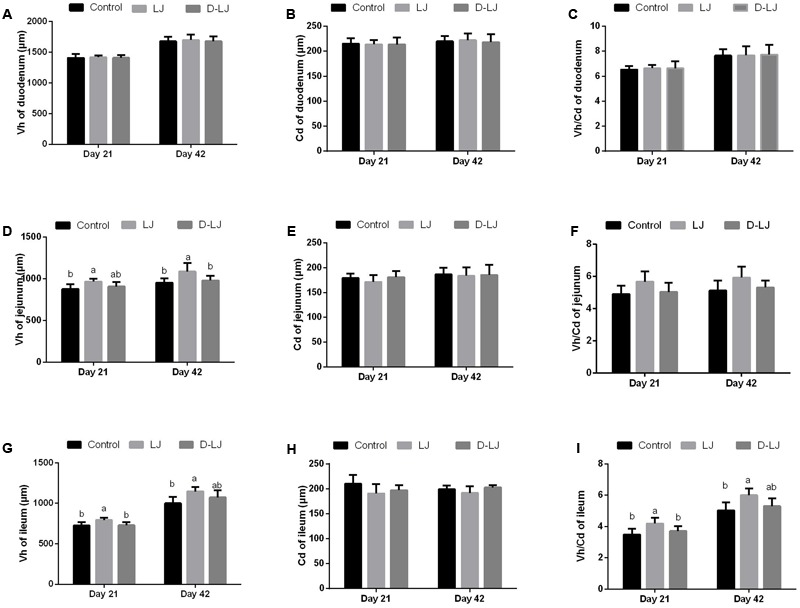
Villus morphology of the small intestine. Bars with different letters are significantly different on the basis of Duncan’s multiple range tests (*P* < 0.05). Data are presented as mean ± standard deviation (six replicates of one chick per cage). **(A–C)** Villus height, crypt depth, and villus height/crypt depth ratio of duodenum; **(D–F)** villus height, crypt depth, and villus height/crypt depth ratio of jejunum; and **(G–I)** villus height, crypt depth, and villus height/crypt depth ratio of ileum.

**FIGURE 5:**
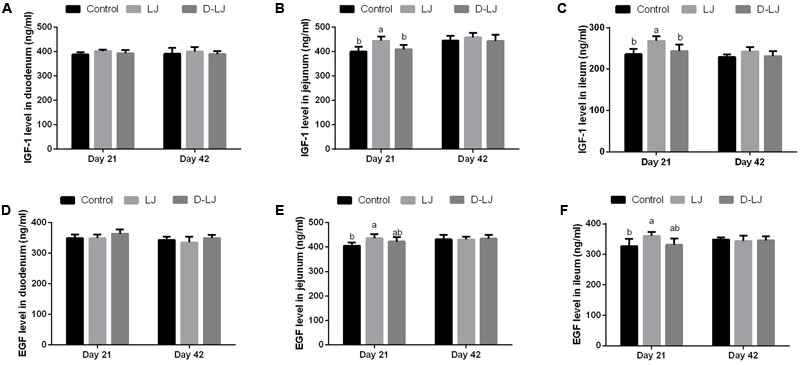
Levels of insulin-like growth factor-1 (IGF-1) and epidermal growth factor (EGF) in the small intestine. Bars with different letters are significantly different on the basis of Duncan’s multiple range tests (*P* < 0.05). Data are presented as mean ± standard deviation (six replicates of one chick per cage). **(A–C)** IGF-1 levels in the duodenum, jejunum, and ileum; **(D–F)** EGF levels in the duodenum, jejunum, and ileum.

The Vh, Cd, Vh/Cd ratio (**Figure 4**), and IGF-1 and EGF levels (**Figure 5**) were evaluated to reveal the effect of LJ on the development of the duodenum, jejunum, and ileum. No significant changes were observed (*P* > 0.05) in the Vh, Cd, and Vh/Cd ratio of the duodenum at both 21 and 42 days (**Figures 4A–C**). A high Vh (*P* < 0.05) was observed for the jejunum in the LJ group at both 21 and 42 days (**Figure 4D**). An increased but limited Vh/Cd ratio (**Figure 4F**) was also observed in the LJ group (*P* = 0.099 at 21 days, *P* = 0.095 at 42 days). However, the Cd of the jejunum (**Figure 4E**) showed no significant change (*P* > 0.05). The Vh and Vh/Cd ratio of the ileum in the LJ group were higher (*P* < 0.05) at 21 and 42 days of age (**Figures 4G,I**). No significant changes were observed (*P* > 0.05) in the IGF-1 and EGF levels in the duodenum at both days 21 and 42 (**Figures 5A,D**) and in the jejunum and ileum at day 42 (**Figures 5B,C,E,F**). In the LJ group, the IGF-1 and EGF levels were higher (*P* < 0.05) in both the jejunum and ileum at day 21, although the EGF level was not significantly higher (*P* > 0.05) than that in the D-LJ group (**Figures 5B,C,E,F**).

### Digestive Enzyme Activities in the Small Intestine

**FIGURE 6:**
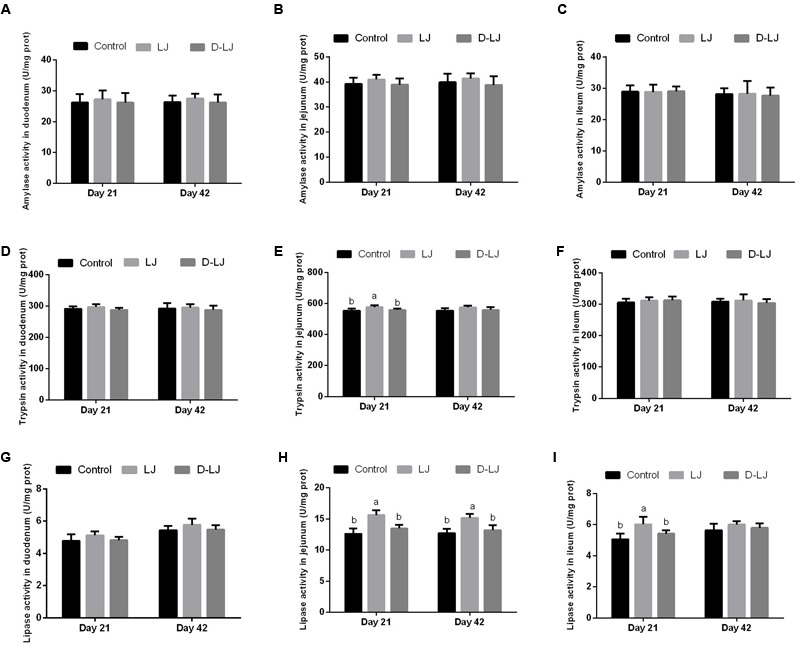
Levels of amylase, trypsin, and lipase activities in the small intestine. Bars with different letters are significantly different on the basis of Duncan’s multiple range tests (*P* < 0.05). Data are presented as mean ± standard deviation (six replicates of one chick per cage). **(A–C)** Amylase activity in the duodenum, jejunum, and ileum; **(D–F)** trypsin activity in the duodenum, jejunum, and ileum; and **(G–I)** lipase activity in the duodenum, jejunum, and ileum.

Results of amylase, trypsin, and lipase activities are shown in **Figure 6**. Significantly higher trypsin and lipase activities in the jejunum and ileum were detected (*P* < 0.05) in the LJ group (**Figures 6E,H**) than those in the control and D-LJ groups at 21 days. Lipase activity of the ileum was also higher (*P* < 0.05) in the LJ group at 42 days (**Figure 6H**). The remaining parameters showed no significant change (*P* > 0.05) among the three experimental groups.

### Microbial Populations Quantified by qPCR

**FIGURE 7:**
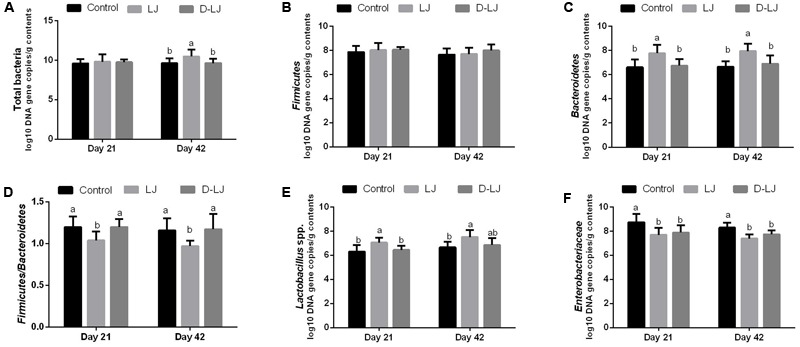
Microbial populations in the ileum as quantified by quantitative PCR. Bars with different letters are significantly different on the basis of Duncan’s multiple range tests (*P* < 0.05). Data are presented as mean ± standard deviation (six replicates of one chick per cage). **(A–C,E–F)** Log_10_ DNA gene copies of total bacteria, Firmicutes, Bacteroidetes, *Lactobacillus* spp., and Enterobacteriaceae. **(D)** Firmicutes/Bacteroidetes ratio.

As shown in **Figure 7**, the population of Bacteroidetes and *Lactobacillus* spp. were significantly increased, whereas that of Enterobacteriaceae was significantly decreased, in the LJ group relative to those in the control group (*P* < 0.05). The Firmicutes/Bacteroidetes ratio was also lower in LJ group (*P* < 0.05). However, there is no difference on the other indexes between D-LJ and the control group (*P* > 0.05). No significant changes were shown on Firmicutes (*P* > 0.05).

## Discussion

A thorough understanding of the mechanisms underlying the direct and indirect effects of a probiotic strain on the host will certainly help improve the efficiency in applying probiotics in the broiler industry. Our recent study demonstrated the ability of *L. johnsonii* BS15 to improve meat quality from poultry by prolonging shelf life and increasing flavor and nutritional content (Liu et al., 2016). The effect of LJ BS15 was characterized by altering parameters related to lipid metabolism, including the fatty acid composition of meat and abdominal fat, which were markedly decreased with increased LJ concentration (Liu et al., 2016). Although lactobacilli exert numerous beneficial effects, such as improved nutrient absorption, vitamin synthesis, and reduced pain perception (Turpin et al., 2010), the exact *in vivo* mechanisms of action of these well-known beneficial bacteria are poorly understood. Hence, the present study aimed to prove the effect of LJ as a probiotic on growth performance and fat deposition. The present work also determined the changes in lipid metabolism, gut microbiota, intestinal development, and digestive ability upon LJ addition to elucidate the mechanisms underlying LJ effects. An appropriate LJ concentration was selected on the basis of a previous study (Liu et al., 2016). Moreover, the D-LJ group was added to determine whether or not the effects of LJ are related to live microorganisms.

Growth performance of the starter and finisher phases were measured in the present study. The enhanced ADG, slightly decreased FCR (*P* > 0.05), and unchanged ADFI at the starter phase suggested that the FCR improvement might be due to improved nutrient digestibility. These results are similar to the findings reported by Shen et al. (2014), who fed broilers with *Lactobacillus plantarum*. Moreover, the FCR was unchanged by LJ at days 22–42 and 1–42, although the ADG and ADFI were both increased. This result indicated that the enhanced ADG was strongly correlated with ADFI and may have contributed to the high body weight at day 22.

As a complicated process involving catalysis by abundant enzymes, lipid metabolism is modulated by numerous hormones. INS is an anabolic hormone that promotes glucose uptake by tissues (Huang et al., 2006). GH decreases lipogenic enzyme activities and adipocyte INS sensitivity. These effects result in reduced lipogenesis and decreased adipose tissue mass (Etherton, 2000). Serum LEP levels are highly related to fat deposition in pigs (Cameron et al., 2000). This marker serves as an indicator of fat content in the human body (Gill et al., 1997). As thyroid hormones, FT3 and FT4 are considered as the main hormones involved in the catabolic processes of lipids (Sheridan and Kao, 1998). The present research showed that LJ exerted no effect on the concentrations of the measured hormones in the sera of broilers. No sign of any kind of hepatotoxicity was observed, as demonstrated by the unaltered serum activities of AST and ALT after 21 and 42 days of LJ supplementation (Huang et al., 2015). The levels of serum lipids and lipoproteins can indicate the metabolic regulations in a steady state, especially the basal adjustment of fatty acid circulation between the liver and the adipose tissue (Mossab et al., 2002). HDLs form a class of lipoproteins with a range of sizes (8–11 nm in diameter). Fatty acids and cholesterol are carried by these lipoproteins from the body’s tissue to the liver (Zhang et al., 2013). Low concentrations of TG and LDL-C but high levels of HDL-C were observed in the LJ group. This observation suggested that LJ altered lipid metabolism. Similar effects were observed by Zhou et al. (2016), who studied *Bacillus licheniformis*. Barter et al. (2010) reported that a link may exist between the changes in HDL-C and TG, but they found no apparent relationship between LDL-C reduction and HDL-C increase. However, Zhang et al. (2011) and Zhao et al. (2013) observed that diet containing *Clostridium butyricum* and *Enterococcus faecium* exerted no effect on the concentrations of TG, TC, HDL-C, and LDL-C in broiler sera. Meanwhile, the serum INS levels were enhanced by *C*. *butyricum*. To date, most studies on lipid metabolism-associated hormones have been performed on mammals, which have different lipid metabolic processes in comparison to birds (Chung et al., 1983; Dunshea et al., 1992; Mossab et al., 2002). Information on the role and action of growth-associated hormones in broilers is also relatively limited. The discrepancy among these studies may be mainly attributed to the different animal and probiotic species investigated.

Consistent with our former study, the present result on abdominal fat percentage indicated that LJ can decrease fat deposition which is considered waste in the poultry industry (Farmani and Rostammiri, 2015; Liu et al., 2016). The result in fat deposition suggested that LJ can promote economic performance by decreasing extra costs. Likewise, a recent study reported the LJ-induced reversal of the trend of obesity in mice (Xin et al., 2014).

Unlike in mammals, LPL mRNA expression in growing chickens is not particularly reactive to aging and nutritional manipulation. This finding indicated the specificity of the physiological response of the broiler chicken LPL (Sato and Akiba, 2002). Chicken LPL is critical in fat accumulation in adipose tissue. The decrease of LPL in adipose tissue through nutritional means may be effective in retarding fatness in broiler chickens (Zimmermann et al., 2004). In the present study, LPL expression in LJ group was repressed at days 21 of age, which may have a relationship with the reduction in fat deposition. Although the ATGL and PPARγ mRNA expression levels in adipose tissue play a significant role in the regulation of fat deposit, these levels remained unchanged in the present study (Youssef and Badr, 2004).

A considerable portion of lipid metabolism occurs in the liver. Thus, the gene expression that alters enzyme capacity in relevant metabolic pathways has attracted significant attention. In this study, all the measured gene expression levels of lipid metabolism-related enzymes, except for ACC, were changed by LJ. This result indicated that LJ can influence both lipid synthesis and catabolism in the liver. SREBP-1c is linked to basic/helix-loop-helix/leucine zipper transcription. This protein can bind to the promoters of several lipogenic enzyme genes and induce their expression (Tabor et al., 1998). SREBP-1c also serves as a lipogenic nuclear transcriptional regulator that can directly affect the expression of ACC, FAS, and SCD1 (Richards et al., 2003). In our study, the expression of ACC exhibited no crucial response. Even so, LJ supplementation significantly inhibited the expression of SREBP-1c and its target genes, FAS and SCD1. FAS is a rate-limiting enzyme in the *de novo* synthesis of fatty acids (Wang and Tian, 2001). SCD1 is an endoplasmic reticulum enzyme that catalyzes the biosynthesis of monounsaturated fatty acids from saturated fatty acids (Cohen et al., 2002). These changes showed certain inhibitory effects caused by LJ on hepatic fatty acid *de novo* synthesis in broilers. Therefore, the reserved SCD1 expression in the present study may have caused low serum TGs and reduced abdominal fat deposition. Research on chickens reported that different diets can change the expression of PPARs in the liver of broilers (Maryam et al., 2011). As a nuclear receptor, PPARα is predominantly observed in the liver; the enzyme facilitates fatty acid catabolism by increasing CPT-1 and ACOX expression. This effect results in the stimulation of mitochondrial and peroxisomal fatty acid β-oxidation (Han et al., 2013). In the present research, LJ supplementation considerably upregulated the gene expression of hepatic PPARα, ACOX, and CPT-1 in the liver and enhanced the fatty acid β-oxidation.

As indicators of intestinal health or morphology, Vh, Cd, and Vh/Cd ratio may be related to intestinal health. Increased Vh may improve nutrient absorption and performance. The Vh/Cd ratio is critical in the evaluation of the developmental state of the intestine (Wu et al., 2013). As a key regulator, IGF-1 is an important regulator that controls long bone growth and energy metabolism. Hence, this growth factor facilitates the growth and function of almost every organ in the body (Giovannucci, 2001). IGF-1 could also increase the Vh in rats (Mantell et al., 1995). EGF, a heat- and acid-stable peptide, along with the EGF receptor, facilitates the proliferation and differentiation of epithelial cells. The two components could significantly enhance gut integrity and heal damaged mucosa or renew epithelial cells (Dvorak, 2010). EGF could also produce different kinds of biologic responses, most of which involve cell replication regulation, cell movement, and cell survival (Harris et al., 2003). In the present study, LJ exerted a positive effect on the jejunum and ileum; LJ increased the Vh and Vh/Cd ratio and the levels of IGF-1 and EGF. This result indicated that LJ could improve the development of the jejunum and ileum. Probiotics can also enhance the growth performance by improving the activities of different digestive enzymes (Timmerman et al., 2006; Fan et al., 2015). Similarly, the present study showed considerably higher activities of trypsin in the jejunum and lipase in both the jejunum and ileum relative to those of the control group.

Numerous animal studies have proved that ingestion of exogenous probiotics has a beneficial effect on growth performance through alteration of the gut microbiota. In chickens, Shen et al. (2014) found that *Lactobacillus plantarum* D22 could enhance the total count of anaerobes, as well as the individual counts of *Lactobacillus* spp. and *Bifidobacterium* spp. Samli et al. (2010) also reported that supplementing *Enterococcus faecium* (3.5 × 10^8^ cfu/kg) positively influenced ileal and cecal microbiota. In the present study, a significantly increased abundance of Bacteroidetes and a low Firmicutes/Bacteroidetes ratio were observed in the LJ group. This result is consistent with the effect of LJ in mice in preventing non-alcoholic fatty liver disease (Xin et al., 2014). Firmicutes and Bacteroidetes can also contribute to host metabolism through several mechanisms, including increased energy harvested from the diet, modulation of lipid metabolism, altered endocrine function, and increased inflammatory response (Greiner and Bäckhed, 2011). A low Firmicutes/Bacteroidetes ratio can be effective against obesity (Ley et al., 2006). Thus, the changes in this study suggested that the low abdominal fat percentage in the LJ group may be attributed to the low Firmicutes/Bacteroidetes ratio. Simultaneously, microbial flora plays a crucial role in inhibiting harmful bacteria and regulating immune responses. In this study, a potentially harmful family of microorganisms was suppressed by LJ BS15. The increased of *Lactobacillus* spp. level could also be indicative of the probable suppression of other non-beneficial bacterial groups (Munita et al., 2012).

Disrupted probiotics can enhance immune responses in broilers. This effect may be caused by a certain amount of bacterial antigens that could stimulate the gastrointestinal immune system (Huang et al., 2004). Some of the parameters in the D-LJ group were slightly improved relative to those in the control group in our study. Significant positive changes in FAS mRNA levels in the liver and Enterobacteriaceae 16S rRNA gene abundance in the ileum were also observed. Lactobacilli-produced exopolysaccharides exert various biological effects that could theoretically confer the host with a range of local and systemic health benefits (Patten and Laws, 2015). These exopolysaccharides may be partly associated with the results in the D-LJ group. Moreover, the enhanced *Lactobacillus* spp. level in the LJ group indicated that LJ may exert its beneficial effects by colonizing the intestinal tract and continuously producing certain metabolites with health benefits. However, whether other factors related to live microbes are involved remains unclear.

Notably, most of the positive influences caused by LJ, including those on growth performance; the mRNA expression levels in the liver and adipose tissue; and the contents of EGF, IGF-1, and digestive enzymes in the small intestine, were noted at day 21. Nevertheless, the serum biochemical parameters and abdominal fat percentage were altered by LJ only at day 42. Hence, altered lipid metabolism-related functional genes and digestive enzymes may result in the manifestation of its related phenotype. A commercial chick exits the egg while harboring a sterile intestinal tract that is fast colonized on the basis of the bacteria present in the environment and not by the bacteria from the hen (Barnes et al., 1980). As a result, the common succession of intestinal colonization is delayed, which enables the exogenous bacteria to be more effective to exert beneficial effects in the chick’s intestinal tract at the starter phase (van der Wielen et al., 2002). Therefore, LJ tended to confer benefits during the starter phase of production.

## Conclusion

Feed supplementation with LJ BS15 may promote growth performance and lower fat deposition in broilers. These changes could be related to an improvement in lipid metabolism in the liver and adipose tissue. Inclusion of LJ BS15 may also enhance intestinal development and digestive ability, as well as help to balance the gut microflora in the small intestine. Based on the results shown in the two production phases and in the D-LJ group, we conclude that live LJ supplementation may provide health benefits by improving lipid metabolism, intestinal development, and gut microflora at the starter phase.

## Author Contributions

HW, DZ, and XN designed the experiments. LL, KP, XQ, and BJ performed the experiments. LL, GL, and ML analyzed the experimental data. HW and DZ wrote this paper. All authors read and approved the final manuscript.

## Conflict of Interest Statement

The authors declare that the research was conducted in the absence of any commercial or financial relationships that could be construed as a potential conflict of interest.
